# Inhibitory Effects of Hwangryunhaedok-Tang in 3T3-L1 Adipogenesis by Regulation of Raf/MEK1/ERK1/2 Pathway and PDK1/Akt Phosphorylation

**DOI:** 10.1155/2013/413906

**Published:** 2013-05-23

**Authors:** Dong Hoon Kwak, Ji-Hye Lee, Dong-Gun Kim, Taesoo Kim, Kwang Jin Lee, Jin Yeul Ma

**Affiliations:** Traditional Korean Medicines- (TKM-) Based Herbal Drug Research, Herbal Medicine Research Division, Korea Institute of Oriental Medicine, Daejeon 305-811, Republic of Korea

## Abstract

Hwangryunhaedok-tang (HRT) has been long used as traditional medicine in Asia. However, inhibitory role of HRT is unclear in early stage of 3T3-L1 adipocyte differentiation related to signaling. In the present study, we investigated the inhibitory effects of HRT on upstream signaling of peroxisome proliferation-activity receptor-**γ** (PPAR-**γ**) and CCAAT/enhancer binding protein-**β** (C/EBP-**β**) expression in differentiation of 3T3-L1 preadipocytes. We found that HRT significantly inhibited the adipocyte differentiation by downregulating several adipocyte-specific transcription factors including PPAR-**γ**, C/EBP-**α**, and C/EBP-**β** in 3T3-L1 preadipocytes. Furthermore, we observed that HRT markedly inhibited the differentiation media-mediated phosphorylation of Raf/extracellular mitogen-activated protein kinase 1 (MEK1)/signal-regulated protein kinase 1/2 (ERK1/2) and phosphorylation of phosphoinositide-dependent kinase 1 (PDK1)/Akt. These results indicate that anti-adipogenesis mechanism involves the downregulation of the major transcription factors of adipogenesis including PPAR-**γ** and C/EBP-**α** through inhibition of Raf/MEK1/ERK1/2 phosphorylation and PDK1/Akt phosphorylation by HRT. Furthermore, high performance liquid chromatography (HPLC) analysis showed HRT contains active antiobesity constituents such as palmatine, berberine, geniposide, baicalin, baicalein, and wogonin. Taken together, this study suggested that anti-adipogenesis effects of HRT were accounted by downregulation of Raf/MEK1/ERK1/2 pathway and PDK1/Akt pathway during 3T3-L1 adipocyte differentiation.

## 1. Introduction

Obesity is characterized at the cellular level by an increase in the number and volume of adipocytes. Adipogenesis is the differentiation process of preadipocytes into adipocytes which play pivotal roles in fat metabolism and closely related to the etiology of obesity and related metabolic disorders [1]. A mouse preadipocyte cell line, 3T3-L1, has been commonly used as an *in vitro* model system to investigate the molecular mechanism of adipogenesis [2]. The 3T3-L1 preadipocytes differentiated into adipocytes and express adipocytes-specific genes in the presence of adipogenic cocktail, containing 3-isobutyl-1-methylxanthine (IBMX), dexamethasone (Dex), and insulin which are commonly abbreviated as MDI [3]. 

Adipocyte differentiation is a complex process that involves coordinated expression of specific genes and proteins associated with each stage of differentiation. Particularly, peroxisome proliferator-activated receptor gamma (PPAR-*γ*) and CCAAT/enhancer binding protein beta (C/EBP-*β*) are key transcription factors of adipogenesis [4]. These two transcription factors induce the expression of adipocytes marker gene such as adiponectin, lipoprotein lipase (LPL), adipocyte fatty acid-binding protein 2 (aP2), and fatty acid synthase (FAS), which are involved in fat metabolism [5, 6]. Moreover, the activation of both extracellular signal-regulated kinase 1/2 (ERK1/2) and Akt pathway would be necessary for adipogenesis [7, 8]. The ERK1/2 is activated by phosphorylation on threonine and tyrosine residues by the dual specificity kinase extracellular mitogen-activated protein kinase 1 (MEK1), which induces the activation of transcription factors involved in growth and differentiation [9]. Several studies have been reported that inhibition of ERK pathway on early stage of differentiation would block adipogenesis, and there are increasing evidences showing the positive role of ERK pathway on adipogenesis. It has been reported that specific and potent MEK-inhibitor U0126, administered during early stage of differentiation, efficiently blocks ERK activity and adipocyte differentiation [10]. Moreover, the Akt signaling pathway is important in transducing the proadipogenic effects of insulin [11] and promotes adipocyte differentiation through increase of PPAR-*γ* expression. Inhibition of Akt pathway in adipocyte differentiation has been shown to inhibit adipogenesis by decrease of PPAR-*γ* expression. 

Recently, medical plant products could provide more effective and economic therapeutic methods for people than ever before. Hwangryunhaedok-tang (HRT) known as huang-lian-jie-du-tang or Orengedokuto is well-known Korean, Chinese, and Japanese traditional herbal medicine consisting of four crude drugs: *Coptis japonica, Phellodendron amurense, Gardenia jasminoides,* and *Scutellaria baicalensis*. This herbal medicine has been used for removing “heat” and “poison” to treat inflammation, cerebrovascular disease, hypertension, gastritis, and liver disease. HRT is also used to clinically prevent or improve obesity [12]. Although the inhibitory effect of HRT on adipocyte differentiation was reported [13], the molecular mechanism for the inhibitory effect of HRT on adipocyte differentiation has not yet been well clarified. Therefore, we investigated the inhibitory effects of HRT on the differentiation of 3T3-L1 preadipocytes and demonstrated that HRT has an inhibitory effect on early stage of MDI-stimulated 3T3-L1 differentiation associated with the downregulation of Raf/MEK1/ERK1/2 and PDK1/Akt pathway.

## 2. Materials and Methods 

### 2.1. Materials

Dexamethasone, insulin, 3-isobutyl-1-methylxanthine, PD98059, and LY294002 were purchased form Sigma Chemical (St. Louis, MO, USA).

### 2.2. Herb Materials and Preparation of HRT

HRT was composed of four medicinal herbs such as *Coptis japonica*, *Phellodendron amurense, Gardenia jasminoides, and Scutellaria baicalensis. *The medicinal herbs were purchased from the Korea Medicine Herbs Association (Yeongcheon, Republic of Korea). The mixture of medicinal herbs including *Coptis japonica *(5 g), *Phellodendron amurense *(5 g)*, Gardenia jasminoides *(5 g)*, and Scutellaria baicalensis *(5 g) was extracted by heating in water for 3-4 h at 90–100°C and prepared in the form of powder by freeze-drying after concentration *in vacuo*. We obtained 3.88 g of HRT powder; 20 mg of HRT powder was dissolved in 1 mL of phosphate-buffered saline (PBS) for treatment on the 3T3-L1 preadipocytes. 

### 2.3. Cell Culture and Adipocyte Differentiation

3T3-L1 preadipocytes (ATCC, Manassas, VA, USA) were cultured in Dulbecco's modified eagle medium (DMEM) high glucose consisting 10% fetal calf serum (FCS) (HyClone, Logan, UT) at 37°C in a humidified atmosphere of 5% CO_2_. The 3T3-L1 cells were differentiated according to a well-established protocol described previously. Briefly, 3T3-L1 cells were cultured in growth media to full confluence for differentiation. Two days after confluence (referred to as day 0), the 3T3-L1 preadipocytes were switched to differentiation media consisting DMEM, 10% fetal bovine serum (FBS), 10 *μ*g/mL insulin, 1 *μ*M Dex, and 0.5 mM IBMX and cultured for 3 days. Next, the cells were maintained in differentiation media containing only 10 *μ*g/mL of insulin, and the medium was changed every 2-3 days. The cells normally differentiated into mature adipocytes on day 7 or 8. 

### 2.4. Real Time Polymerase Chain Reaction (RT-PCR)

Total cellular mRNA was extracted using Easy-Blue reagents (Invitrogen, Grand Island, NY, USA) according to instructions provided by the supplier. Quantity and quality of isolated mRNA were assessed using nanodrop spectrophotometer (Thermo scientific, Ltd.), and samples were processed for cDNA synthesis using cDNA synthesis kit (Invitrogen, Grand Island, NY, USA). The cDNA synthesis was carried out at 37°C for 1 h using a Veriti 96-well thermal cycler (Applied Biosystems, USA). The PCR conditions were as follows: 25 cycles of 94°C for 30 s, 55°C for 30 s, and 68°C for 30 s. The amplified products were separated by electrophoresis on 1.5% agarose gels. RT-PCR analysis used the sequences of primers including PPAR-*γ*, C/EBP-*β*, C/EBP-*α*, aP2, LPL, adiponectin, and stearoyl-CoA desaturase-1 (SCD-1), 18s (Table 1). 

### 2.5. *In Vitro* Cytotoxicity Assay

Preconfluent 3T3-L1 preadipocytes (1 × 10^4^ cells/well) were maintained in 96-well culture plate for 72 h in presence of HRT (0–2000 *μ*g/mL) and standard compounds including wogonin, geniposide, palmatine, baicalein, berberine, and baicalin (0–1000 *μ*M) of vehicle (PBS). At the end of incubation period, 10 *μ*L of MTT (5 mg/mL in PBS) was added to wells and the plate was incubated at 37°C for 4 h. At the end of incubation, culture media were discarded and the wells were washed with PBS. Later, 150 *μ*L of dimethyl sulfoxide (DMSO) was added to all the wells and incubated for 30 min at room temperature with constant shaking. Absorbance was read at 540 nm using ELX800 Universal Microplate Reader (Bio-Tek instruments, Inc., Winooski, VT, USA) and subsequently percentage (%) cell viability was calculated.

### 2.6. Qualitative Analysis of Adipocyte Differentiation

On day 4 or 8, cells were stained with Oil red O. For Oil red O staining, cells were washed with PBS and stained with filtered Oil red O solution for 10–30 min. After staining the lipid droplet the Oil red O staining solution was removed, and the plates were rinsed with water and dried, and photographs were taken in Nikon inverted microscope using Nikon digital camera system. In another set of experiment, the stained adipocytes were treated with 100% isopropanol (to extract intracellular Oil red O stain) and the absorbance (optical density, OD) was read at 520 nm. Percentage adipogenesis was calculated as OD of treated cells/OD of untreated cells × 100. 

### 2.7. Protein Extraction and Western Blotting

Cultured and differentiated cells were harvested using a cell scraper and lysed with ice-cold Pro-PREPTM buffer (INtRON, USA). The cell lysates were centrifuged at 14000 rpm for 20 min at 4°C to remove insoluble materials. The protein concentrations were determined using a BCA protein assay kit (Pierce, Rockford, IL). Protein extracts was ruined in 10% SDS-polyacrylamide gel and transferred to nitrocellulose membranes at 150 mA for 1 h. The membrane was then blocked for 1 h at room temperature with PBS containing 5% bovine serum albumin (BSA) and incubated with 1 : 1000 dilutions of primary antibodies (anti-Akt, anti-pAkt, anti-pPDK1, anti-pRaf, anti-ERK1/2, anti-pERK1/2, anti-Ras, anti-pMEK1, anti-PPAR-*γ*, anti-CEBP-*α*, anti-CEBP-*β*, *β*-actin, and anti-Raf (Cell Signaling, Beverly, MA, USA)) overnight at 4°C and subsequently with a horseradish peroxidase-conjugated anti-rabbit secondary antibody (diluted 1 : 1000, Cell Signaling, Beverly, MA, USA) for 1 h at room temperature. Peroxidase activity was visualized using the ECL kit (Thermo, USA). 

### 2.8. Chromatographic Conditions

HRT powder and standard compounds (geniposide, wogonin, baicalein, berberine-HCl, palmatine-HCl, and baicalin) were accurately weighed and dissolved in 60% methanol. They were filtered (using a 0.45 *μ*m membrane filter) and stored at 4°C before high performance liquid chromatography (HPLC) analysis. The HPLC diode array detector (DAD) system (Hitachi Co., Tokyo, Japan) consisted of a pump (L-2130), column oven (L-2300), diode array UV/VIS detector (L-2455), and autosampler (L-2200). System control and data analysis were performed using EZchrom Elite software for Hitachi. The analysis of HRT and standard compounds was conducted using a Phenomenex C18 column (5 *μ*m, 4.6 mm × 250 mm). The mobile phase consisted of water with acetonitrile and 0.1% trifluoroacetic acid (TFA) at a flow rate of 1.0 mL/min, and the column temperature was maintained at 30°C. The elution conditions applied were 0–5 min, isocratic 20% B; 5–25 min, linear gradient 20%–30% B; 25–35 min, linear gradient 30%–35% B; 35–45 min, linear gradient 35%–40% B; 45–55 min, linear gradient 40%–35% B. 

### 2.9. Preparation of the Standard Solution

**Figure 1:**
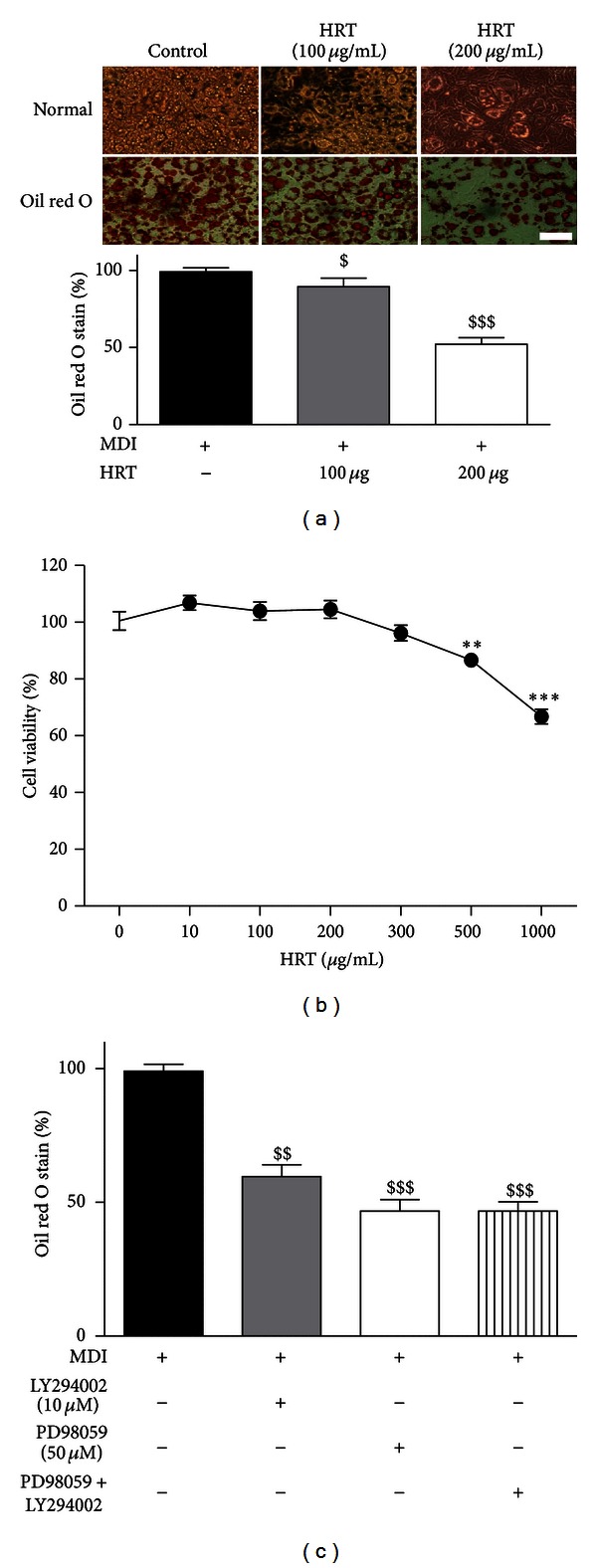
HRT inhibits adipocyte differentiation of 3T3-L1 cells. Two-day postconfluent 3T3-L1 preadipocytes (day 0) were treated with the indicated concentrations of HRT extract and were replete every 2 days along with relevant media cocktail up to day 8. Cells treated with 1X PBS were used as control. (a) Intracellular lipids were stained with Oil red O. (b) Cell viability was determined by MTT assay. (c) Inhibition of signaling (Erk and Akt) inhibitors (PD98059 and LY294002) in accumulation of intracellular lipids. The results were confirmed by three independent experiments, which were each conducted in triplicate. Data are expressed as the mean ± S.D. **P < 0.01 versus controls (cell viability), ***P < 0.001 versus controls (cell viability), ^$^ *P* < 0.05 versus control, and ^$$$^ *P* < 0.001 versus control (Oil red O). Bar indicates 100 *μ*m.

The standard solution of six such as geniposide (1), baicalin (2), palmatine (3), berberine (4), baicalein (5), and wogonin (6) was prepared by dissolving 2 mg of each compound in 10 mL of methanol and adjusting the concentration to 200 ppm. From the results, Table 1 shows the identification and quantitative analysis of useful components in the HRT. Geniposide, baicalin, palmatine, berberine, baicalein, and wogonin were detected at 254 nm, respectively. Also, the profile of main components, geniposide (1, *t*_*R*_: 17.39 min), baicalin (2, *t*_*R*_: 36.02 min), palmatine (3, *t*_*R*_: 39.54 min), berberine (4, *t*_*R*_: 39.75 min), baicalein (5, *t*_*R*_: 48.29 min), and wogonin (6, *t*_*R*_: 58.33 min), was identified in HRT (Figure 1). Particularly, the extraction efficiency of berberine (19.08 ± 0.506 *μ*g) in HRT was the highest.

### 2.10. Statistical Analysis

All values in the figures of the present study indicate means ± standard deviation (S.D.), and all determinations were repeated three times. The one way analysis of variance (ANOVA) was used to evaluate the difference among multiple groups and the independent sample *t*-test for difference between two treatment groups. The data were analyzed using GraphPad Prism software (GraphPad Software Inc., Chicago, IL, USA), and *P* < 0.05 was assessed as statistically significant. 

## 3. Results

### 3.1. Effects of HRT on 3T3-L1 Adipocyte Differentiation and Cell Viability

To examine the effects of HRT on adipocyte differentiation, 3T3-L1 cells were grown in differentiation medium containing MDI for 8 days in the presence or absence of 100 or 200 *μ*g/mL of HRT. To assay for adipocyte differentiation, Oil red O staining was used to measure lipid droplets. When the cells were incubated with differentiation media, lipid droplets were accumulated, but the accumulation of lipid droplet was significantly decreased by treatment with HRT (Figure 1(a)). Viability of 3T3-L1 cells was not significantly affected at concentration below the 300 *μ*g/mL of HRT (Figure 1(b)). These results indicate that the HRT significantly suppressed lipid droplet accumulation in 3T3-L1 cells and that inhibitory effects of HRT was not due to cytotoxic.  Figure 1(c) indicated the inhibitory effects of inhibitors such as PD98059 (inhibitor of Erk phosphorylation, 50 *μ*M) and LY294002 (inhibitor of Akt phosphorylation, 10 *μ*M) in 3T3-L1 cells differentiation. 

### 3.2. HRT Inhibits the mRNA and Protein Level of Transcription Factors and Adipocyte Markers in Adipogenesis

**Figure 2:**
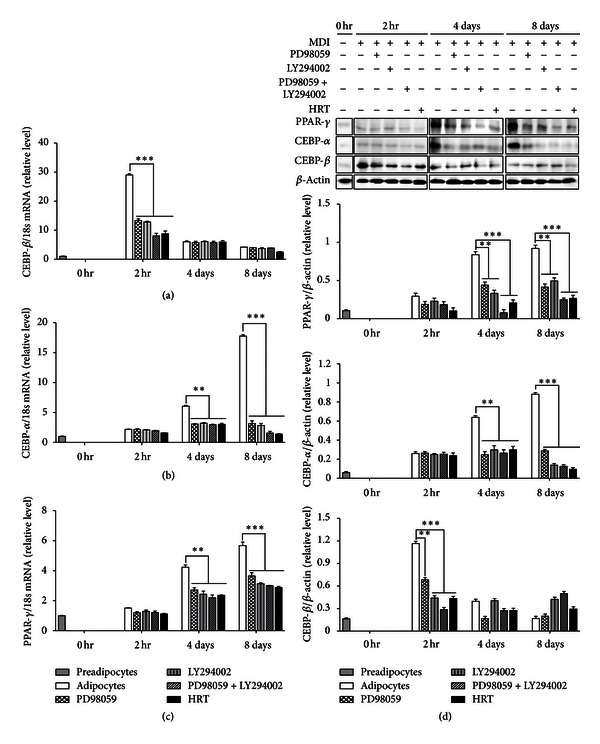
HRT inhibits the expression of transcription factors in differentiation of 3T3-L1 cells. Preadipocytes were induced to differentiate with extract of HRT (200 *μ*g/mL) and harvest at 2 h, day 4, and day 8 during the differentiation period. Panels are (a) the mRNA of C/EBP-*β*, (b) the mRNA of C/EBP-*α*, and (c) the mRNA of PPAR-*γ*. Panel (d) indicates the protein expression level of major factors. Values are mean ± S.D. of data from three separate experiments; each experiment was performed in triplicate. **P < 0.05 and ***P < 0.001 versus control. Treatment of PD98059 (50 *μ*M) and LY294002 (10 *μ*M) is positive control for HRT treatment.

**Figure 3:**
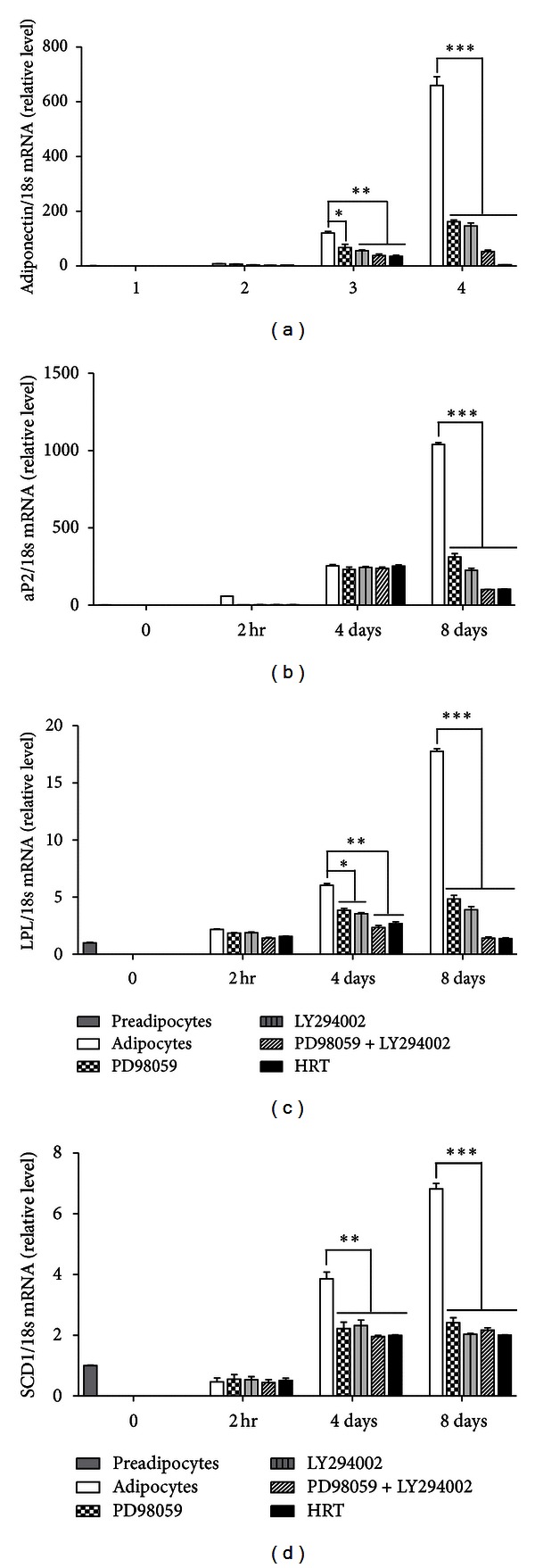
HRT inhibits the expression of adipocyte-specific genes in differentiated 3T3-L1 cells. Preadipocytes were induced to differentiate with extract of HRT (200 *μ*g/mL) and harvest at 2 h, day 4, and day 8 during the differentiation period. Panels are (a) the mRNA of adiponectin, (b) the mRNA of aP2, (c) the mRNA of LPL, and (d) the mRNA of SCD-1. Values are mean ± S.D. of data from three separate experiments; each experiment was performed in triplicate. **P < 0.005 and ***P < 0.001 versus control. Treatment of PD98059 (50 *μ*M) and LY294002 (10 *μ*M) is positive control for HRT treatment.

Adipogenesis process is accompanied by increase of adipogenic transcription factors and adipocyte-specific genes expression. Particularly, PPAR-*γ* and C/EBP-*α* are master adipogenic transcription factors. These transcription factors are induced during adipogenic differentiation of 3T3-L1, which are regulated by C/EBP-*β* at early stage. To address the effect of HRT on adipogenic transcription factors expression, mRNA expression levels of PPAR-*γ* and C/EBP family (C/EBP-*α* and C/EBP-*β*) were measured during differentiation of 3T3-L1 cells in the presence or absence of HRT and signaling inhibitors (PD98059 and LY294002) together with MDI (Figures 2(a)–2(c)). An increase in C/EBP-*β* mRNA in response to MDI was detectable at 2 hours, but HRT, ERK inhibitor (PD98059), and Akt inhibitor (LY294002) treatment significantly inhibited the increase of C/EBP-*β* mRNA (Figure 2(a)). The mRNA levels of C/EBP-*α* and PPAR-*γ* were also significantly decreased in induction of adipogenesis after 4 and 8 days (Figures 2(b) and 2(c)). Major proteins expression of adipocytes differentiation was measured during differentiation of 3T3-L1 cells (Figure 2(d)). Patterns of adipogenic protein expression were similar to the patterns of adipogenic mRNA expression (Figure 2(d)). Interestingly, HRT treatment also led to the suppression of PPAR-*γ* target genes expression, including aP2, LPL, adiponectin, and SCD-1 (Figure 3). These results suggested that HRT inhibits adipogenesis through reducing the expression of C/EBP-*β* that leads to downregulation of the expression of C/EBP-*α* and PPAR-*γ*. 

### 3.3. HRT Inhibits the Phosphorylation of Raf/MEK1/ERK1/2 Pathway and PDK1/Akt Pathway in Differentiation of 3T3-L1 Cells

**Figure 4:**
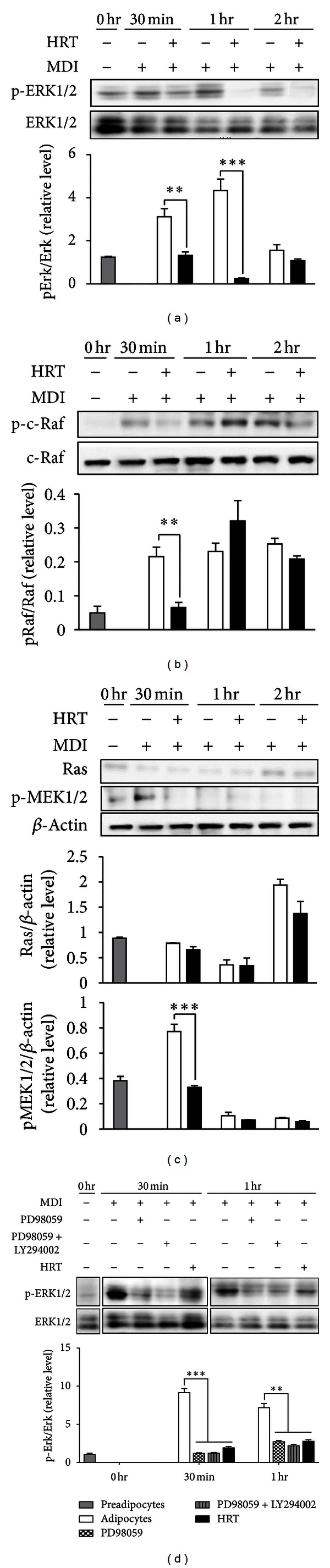
HRT inhibits the Raf/MEK/ERK1/2 phosphorylation in early stage of 3T3-L1 adipocytes differentiation. Preadipocytes were induced to differentiate with HRT (200 *μ*g/mL) and harvest at 30 min, 1 h, and 2 h and during the early stage of differentiation. Panels are indicating (a) the expression of ERK1/2, (b) the expression of c-Raf, and (c) the expression of Ras/MEK1. Panel is indicating (d) treatment of Erk phosphorylation inhibitor (PD98059, 50 *μ*M) as a positive control for HRT treatment. The proteins were analyzed by western blot. Values are mean ± S.D. of data from three separate experiments; each experiment was performed in triplicate. **P < 0.01 versus control, and ***P < 0.001 versus control.

**Figure 5:**
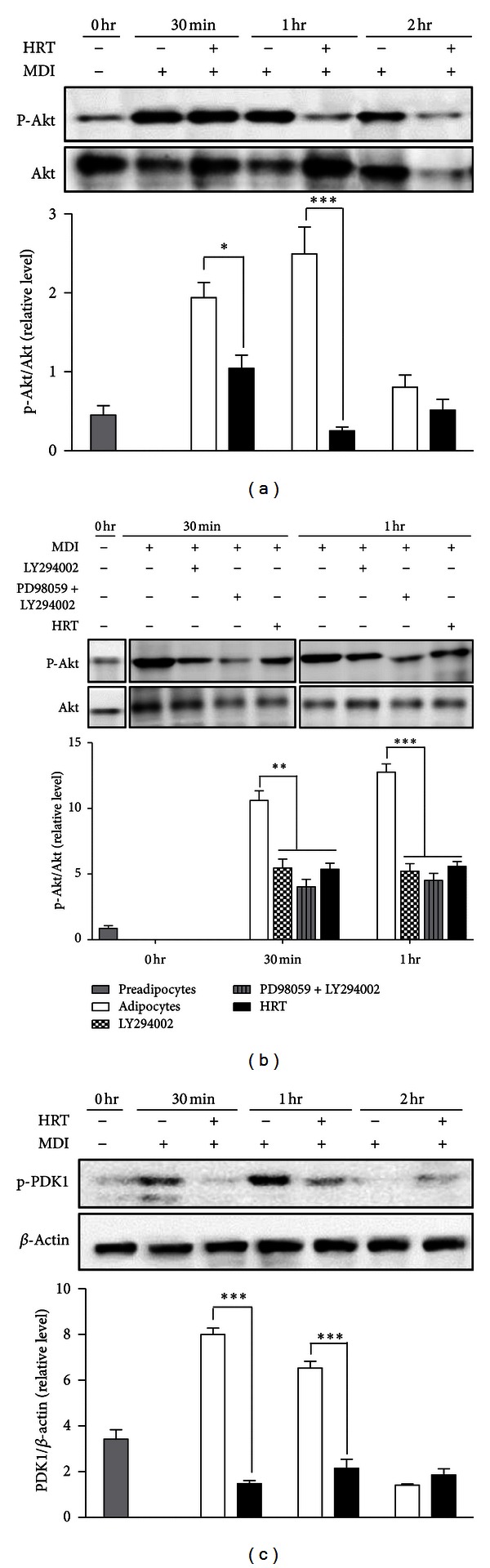
HRT inhibits the PDK1/Akt phosphorylation during differentiation of 3T3-L1 cells. Preadipocytes were induced to differentiate with HRT (200 *μ*g/mL) and harvest at 30 min, 1 h, and 2 h during the early stage of differentiation. Panel (a) indicates the phosphorylation of Akt. Panel (d) is indicating treatment of Akt phosphorylation inhibitor (LY294002, 10 *μ*M) as a positive control for HRT treatment. Panel (c) indicates the activation of PDK1. The proteins were analyzed by western blot. Values are mean ± S.D. of data from three separate experiments; each experiment was performed in triplicate. *P < 0.05 versus control, and ***P < 0.001 versus control.

One of the established signaling mechanisms for increasing PPAR-*γ* gene expression is mediated by ERK1/2 and Akt in adipocyte differentiation [7]. Therefore, we investigated the effect of HRT on ERK- and Akt-mediated signaling pathways. HRT effectively suppressed MDI-induced phosphorylation of ERK and its upstream signals, such as c-Raf and MEK1 (Figures 4(a) and 4(b)). The quantitative data indicated that the protein expressions of p-ERK in MDI was treated with HRT at 30 min and 1 h were 42.6% and 5.5% lowered, respectively, compared with that in the controls without HRT treatment. However, Ras was not significantly affected in 3T3-L1 differentiation at after 30 min, 1 h, and 2 h (Figure 4(c)). As shown in Figure 4(d), inhibitor of Erk phosphorylation (PD98059) treatment induced the significant inhibition of Erk phosphorylation, as a positive control for HRT treatment. Akt pathway has been shown to regulate adipocyte differentiation by modulation of PPAR-*γ* expression [14]. In this study, we found that HRT significantly decreased phosphorylation of Akt compared with control (Figure 5(a)). Since Akt phosphorylation was mediated by phosphorylation of phosphoinositide-dependent kinase 1 (PDK1) which is a main mediator upstream of Akt phosphorylation, we also addressed the effect of HRT on PDK1 phosphorylation. As shown in Figure 5(b), as a positive control for HRT treatment, inhibitor of Akt phosphorylation (LY294002) treatment more strongely inhibited the p-Akt expression. Phosphorylation of PDK1 was significantly inhibited compared with control during adipogenesis early stage by HRT (Figure 5(c)). The results suggested that the inhibition of adipocyte differentiation by HRT was associated with the regulation of upstream of ERK1/2 and Akt pathway. 

### 3.4. HPLC Analysis

**Figure 6:**
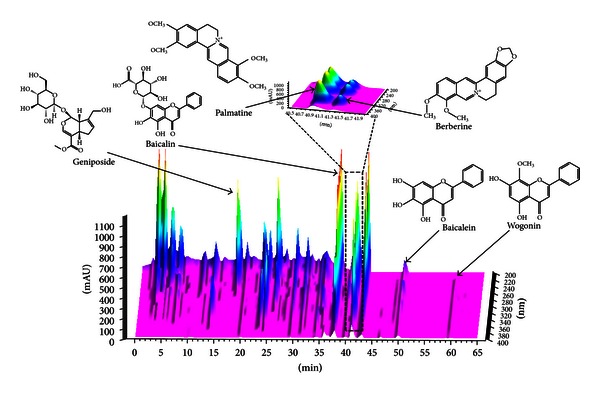
Three-dimensional HPLC chart of HRT major constituents. HPLC profiling of standard components contained in HRT. The HPLC chromatogram of components was monitored at 230 nm.

To confirm and quantify the six standard contents of HRT, HPLC analysis was optimized and performed (Figure 6). The analysis was carried out using a C_18_ column, and flow rate of mobile phase was fixed at 1.0 mL/min. The gradient elution proportions of water and acetonitrile were tested to achieve desired separation. Trifluoroacetic acid was added to water in order to improve a peak shape and inhibit peak tailing. The UV wavelength of six components was set based on maximum UV spectra absorption of each component. Geniposide, palmatine, berberine, baicalin, baicalein, and wogonin were detected at 280 nm and glycyrrhizin was detected at 254 nm. The amount of six components was calculated using a calibration curve of each component. From the results, Table 2 shows main components identified in HRT. The characterization of each component was conducted by comparing retention time and UV spectrum of target peaks in HRT. The profiles of main components were shown in Figure 6.

### 3.5. Effects of Major Six Compounds of HRT on 3T3-L1 Adipocyte Differentiation

**Figure 7:**
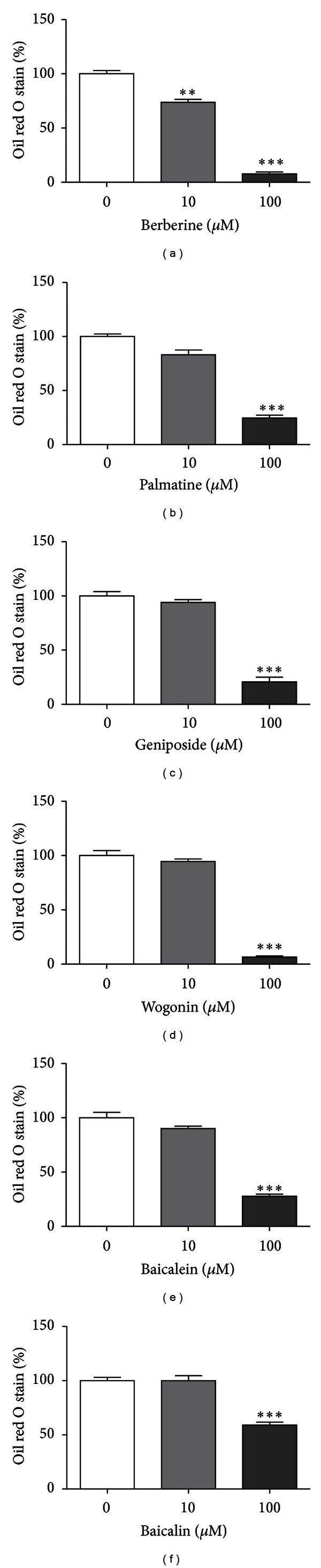
Inhibitory effect of major constituents of HRT in adipocyte differentiation of 3T3-L1 cells. 3T3-L1 preadipocytes were treated with or without major constituents of HRT (0–500 *μ*g/mL) during differentiation for 8 days. Oil red O staining of 3T3-L1 cells treated with (a) berberine, (b) palmatine, (c) geniposide, (d) wogonin, (e) baicalein, and (f) baicalin was determined. Each value was expressed as a percentage of the differentiated control (100%). The data show the mean ± S.D. of three experiments. Oil red O: **P < 0.01 versus control; ***P < 0.001 versus control.

We investigated the inhibitory effect of six chemicals, including berberine, palmatine, geniposide, baicalein, wogonin, and baicalin on adipocyte differentiation. Fascinatingly, six major compounds (berberine, geniposide, palmatine, wogonin, baicalin, and baicalein) decreased intracellular TG accumulation at concentrations from 100 *μ*M, compared with control (Figures 7(a)–7(f)). Particularly, berberine reduced intracellular lipid droplet accumulation at concentrations from 10 to 100 *μ*M, compared with the differentiated control (Figure 7(a)). These results indicate that the inhibitory effect of HRT (berberine, palmatine, geniposide, baicalein, baicalin, and wogonin) on TG accumulation was maybe due to major six compounds of HRT. 

## 4. Discussion

Adipocytes are endocrine cells for energy storage, and adipogenesis is an important process for maintaining physiological cellular function [15, 16]. 3T3-L1 cells were differentiated to adipocyte with the accumulation of intracellular lipid under adequate *in vitro* culture condition. Previous studies showed that many natural compounds including genistein, esculetin, resveratrol, guggulsterone, conjugated linoleic acid, capsaicin, and procyanidins inhibited adipogenesis [17, 18]. In this study, we demonstrate that HRT significantly inhibited 3T3-L1 preadipocytes differentiation through downregulation of ERK1/2 and Akt pathway. 

In the present study, we found that accumulation of TG droplet was significantly decreased by HRT treatment dose dependently without cytotoxicity (Figure 1(a)). Since HRT contains four constituents such as *Coptis japonica, Phellodendron amurense, Gardenia jasminoide,* and *Scutellaria baicalensis*, we confirmed the safety of HRT used in this study based on the toxicity experiment of *in vitro* MTT assay.

The differentiation of preadipocytes is regulated by a complex regulation of transcription factors [19]. The main transcription factors are nuclear receptor PPAR-*γ* and members of C/EBPs, which are important for the process of adipocyte differentiation [20]. PPAR-*γ* has been shown to be sufficient and necessary for adipogenesis [21, 22]. PPAR-*γ* is also known for binding to C/EBP-*α* promoter region that induces the expression of C/EBP-*α*, which was regulated by C/EBP-*β* in adipocyte differentiation [23]. C/EBP-*β* was expressed earlier than both C/EBP-*α* and PPAR-*γ* during adipogenesis [24] and acted to induce the expression of C/EBP-*α* and PPAR-*γ* [5, 25]. We found that HRT considerably reduces the mRNA level and the protein level of C/EBP-*β* during adipogenesis (Figures 2(a) and 2(d)) and that it significantly inhibited the expression of C/EBP-*α* and PPAR-*γ* genes and proteins (Figures 2(b)–2(d)). These results suggested that HRT reduced the expression of C/EBP-*α* and PPAR-*γ* genes occur dependently to the C/EBP-*β* gene expression during adipogenesis. 

It has been reported that the downstream target genes of PPAR-*γ* and C/EBP-*α* such as LPL, adiponectin, SCD-1, and aP2 are adipocyte-specific genes involved in maintaining adipocytes [5, 6], and the expression of these adipocyte-specific genes was regulated by PPAR-*γ* and C/EBP-*α* [26, 27]. In our study, HRT was able to repress expression of adipocyte-specific genes such as adiponectin, aP2, FAS, and LPL (Figures 3(a)–3(d)). These results supported our hypothesis that the HRT inhibited the expression of major transcription factors during adipogenesis. 

Activation of ERK pathway during adipogenesis promoted differentiation by activating the factors that regulated PPAR-*γ* and C/EBP-*α* expression [7]. Moreover, it was reported that ERK1/2 was upstream of PPAR-*γ* and C/EBP-*α*, and reduction of ERK1/2 phosphorylation induced expression of PPAR-*γ* and C/EBP-*α* [28]. In this study, we observed that the HRT significantly inhibited the phosphorylation of ERK1/2 in adipogenesis and the phosphorylation of upstream mediators of ERK1/2 including MEK1 and Raf (Figures 4(a)–4(d)). However, expression of Ras has no appreciable differences compared with control (Figure 4(c)). These results indicated that HRT induced the inhibition of adipogenesis through the reduction of MEK1/ERK1/2 phosphorylation by regulation of Raf phosphorylation. 

Akt was another important mediator in adipocyte differentiation, and activation of Akt could induce the differentiation of 3T3-L1 preadipocyte [29, 30]. Several studies reported that mouse embryonic fibroblasts (MEFs) lacking Akt failed to differentiate into mature adipocytes [31], and an RNA-mediate decreased of Akt was found to block the differentiation of 3T3-L1 preadipocyte [32]. Phosphorylation of Akt was regulated by PDK1 as an upstream kinase of Akt [33]. We also evaluate the effect of HRT on upstream signaling of PPAR-*γ* and C/EBP-*α* and found that the HRT significantly inhibited the phosphorylation of Akt in adipogenesis (Figures 5(a) and 5(b)) and the phosphorylation of upstream mediator of Akt such as PDK1 (Figure 5(c)). These results suggested indicated that HRT induced the inhibition of adipogenesis trough regulation of Akt phosphorylation by inhibition of PDK1 phosphorylation. 

As shown in Figure 6, we identified six main compounds (berberine, palmatine, geniposide, baicalein, wogonin, and baicalin) in HRT using HPLC analysis and observed that six main components of HRT inhibited the TG accumulation in 3T3-L1 adipocytes (Figure 7). Consistent with our results, a previous study reported inhibitory effects of berberine on TG accumulation of 3T3-L1 adipocyte by PPAR-*γ* [34]. Additionally, previously, studies reported that baicalein and baicalin inhibit the adipocyte differentiation [35, 36]. Other studies indicated that baicalin inhibits PDGF-BB-stimulated vascular smooth cell proliferation by suppressing ERK signaling [37], and baicalein attenuates intimal hyperplasia after rat carotid balloon injury through inhibiting ERK, and Akt in vascular smooth muscle cells [38]. It was also reported that berberine reduced ERK activity in melanoma cells [39]. These facts suggest that the inhibitory effects of HRT on ERK and Akt pathway in early stage differentiation of adipocytes might be related to active components contained in HRT including berberine, wogonin, geniposide, baicalin, and baicalein as shown in Figure 7. 

## 5. Conclusion

Antiadipogenesis mechanism of HRT involves the downregulation of the major transcription factors of adipogenesis including PPAR-*γ* and C/EBP-*α* by inhibition of Raf/MEK1/ERK1/2 and PDK1/Akt phosphorylation and resultant downregulation of lipid metabolizing enzymes which are involved in the transport, uptake, and synthesis of lipids needed for the accumulation of lipid in adipocytes. These study results demonstrated that antiadipogenic effects of HRT in differentiation of 3T3-L1 preadipocytes are the regulation of Raf/MEK1/ERK1/2 pathway and PDK1/Akt pathway. 

## Figures and Tables

**Table 1:** Sequences of primers used for RT-PCR.

Gene name	Forward primer	Reverse primer
PPAR-*γ*	TCACAAGAGGTGACCCAATG	CCATCCTTCACAAGCATGAA
C/EBP-*β*	GTTTCGGGAGTTGATGCAATC	AACAACCCCGCAGGAACAT
C/EBP-*α*	GTGTGCACGTCTATGCTAAACCA	GCCGTTAGTGAAGAGTCTCAGTTTG
aP2	CCAATGAGCAAGTGGCAAGA	GATGCCAGGCTCCAGGATAG
LPL	GGCCAGATTCATCAACTGGAT	GCTCCAAGGCTGTACCCTAAG
Adiponectin	GGAGATGCAGGTCTTCTTGGT	TCCTGATACTGGTCGTAGGTGAA
SCD-1	CCGGAGACCTTAGATCGA	TAGCCTGTAAAAGATTTCTGCAAACC
18s	CATTCGAACGTCTGCCCTATC	CCTGCTGCCTTCCTTGGA

**Table 2:** Identification and quantitative analysis of useful components from Hwangryunhaedok-tang.

	Item	Geniposide	Baicalin	Palmatine	Berberine	Baicalein	Wogonin
HRT	Retention time (*t*_*R*_, min)	17.39	36.02	39.54	39.75	48.29	58.33
	Amount (*μ*g^a^)	15.71 ± 0.08	8.72 ± 0.41	1.10 ± 0.03	19.08 ± 0.51	3.98 ± 0.02	3.50 ± 0.01

^a^unit: *μ*g; loading amount = 1 mg of sample.
